# Capitalistic Chemistry

**DOI:** 10.1021/acs.jpcc.5c00660

**Published:** 2025-03-05

**Authors:** Lars G.M. Pettersson

**Affiliations:** Department of Physics, AlbaNova University Center, Stockholm University, 106 91 Stockholm, Sweden

## Abstract

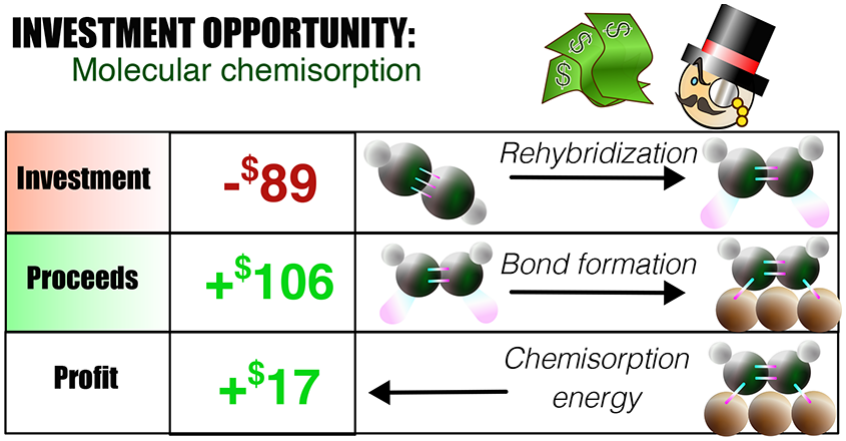

The concept of “bond strength” is of essence for modeling every kind of reactive chemistry. Particularly within the field of catalysis and surface science, the interaction strength of adsorbates to surfaces affects the activity, selectivity, and stability of intermediates and transition states. Here, we introduce a simple approach to chemical reactions through an analogy with business. We regard rehybridization as the *investment* a molecule makes to prepare its electronic and geometrical structure to form new bonds. The resulting bond strength is the total *proceeds* from bond formation, and the difference (exothermicity) is the *profit*. The predictive power lies in the fact that any change in the electronic structure to prepare for bond formation requires the involvement of specific excited states. Thus, with knowledge of the energy needed for this excitation (investment) and the strength of the resulting interaction one can predict whether a specific reaction or bonding mode will be favored. We apply this concept to rationalize observed binding modes at surfaces and the often observed large structural changes even for “weakly” chemisorbed systems and finally to justify using small metal clusters to correct chemisorption energies from periodic DFT calculations.

## 1 Introduction

In quantum chemistry, the basis set defines the maximum number of mutually orthogonal wave functions that can be constructed to describe the ground and excited states in the calculation. Each has a specific energy and character, and the variational principle determines the optimum mixture to minimize the energy; involving higher-energy states comes at a cost that must be offset by a higher gain. Similarly, in periodic boundary calculations on extended systems the size of the unit cell, *k*-point sampling, and energy cutoff define the variational space. The Bloch states are orthogonal and could, in principle, be used to construct a sequence of orthogonal wave functions based on occupancy below and above the Fermi level. Albeit impractical, this is implicit when calculating band gaps or building analyses on the density of states below and above the Fermi level, including how far up in energy the unoccupied states lie. Altogether, starting from an isolated molecule or pristine metal surface, the unoccupied states provide the computational flexibility to describe changes in the electronic density due to bond formation or response to a different environment. Importantly, any change from one state to another requires mixing the initial wave function with what were excited states in order to arrive at the new ground state, e.g., form a new bond.

For the real molecule, the “basis set” consists of the available electronic excited or charge-transfer states, where their character and energy determine their availability to contribute to a specific bonding situation. Thus, knowledge of the expense to reach the excited state that is involved in the bonding provides the rehybridization cost, or *investment*, that is required to change the electronic and geometrical structure. Knowledge of the strength of the resulting bond, i.e., *return or total proceeds*, from this investment gives the chemisorption or reaction energy as the difference, i.e., *profit*, between the investment in rehybridization and what is returned from the bond formation (all the energy gained relative to the gas phase rehybridized state). Thus, knowledge of the excitation energy and characteristic bond strength allows assessing the feasibility of a specific bonding mode, i.e., if the resulting bonds are sufficient to overcome the necessary investment in rehybridization. Similarly, the spectrum of the molecule together with measured reaction energy provides an estimate of the interaction energy or bond strength. This view on chemisorption energies or reaction exothermicity directly shows that it can be misleading to use the magnitude of the resulting binding energy as a measure of the strength of an interaction or to distinguish between chemisorption and physisorption. A small value can be due to a weak interaction with small investment in rehybridization or to a strong bond, offset by a large investment to reach the bonding state.

The main strategy is exemplified by the pioneering studies by Carter and Koel.^1−3^ A σ-bond between carbon in a hydrocarbon and Pt (in Pt(111)) was estimated to 53 kcal/mol based on computed energetics and used to distinguish between reaction mechanisms in the decomposition of ethylene on Pt(111).^1^ This methodology was further extended into a systematic approach to extract bond energies, building on the analysis of the bonding in terms of bond character and which gas-phase electronic states contribute. Applications have been made to, e.g., dehydrogenation of cyclohexane to benzene on Pt(111).^2^ A similar estimate of ∼50 kcal/mol was obtained for carbon bonding to copper in unsaturated hydrocarbons on Cu surfaces by Triguero et al.^4^ by explicitly taking into account the π → π* excitation energy to reach the bond-prepared diradical state that can form the two σ-bonds required for a lying-down geometry.

By comparing the energy cost to reach the involved adsorbate excited states in the gas phase with the gain from bond formation, “capitalistic chemistry” is also predictive in terms of possible bonding schemes of an adsorbate to a surface. For CO and N_2_, the cost for this excitation is too high to be offset by two σ-bonds, resulting in vertical chemisorption with only partial involvement of the π → π* excitation unless additional interactions are available.^5^

King^6^ and Campbell and co-workers^7−10^ have reported very accurate chemisorption energies through single-crystal adsorption calorimetry,^11^ but to convert these to bond energies requires knowledge of the electronic states that are involved in the reacting molecule. Here, bond strengths determined from theory or known experimental excitation energies have been applied.^7^ In addition, the energy cost of changes in the substrate, e.g., surface reconstruction, segregation, or electronic structure, needs to be included in the account.

In the following, I will review several earlier applications of this type of analysis of bonding and reactivity with the aim to provide a simplified, intuitive, and unified view of bond formation in terms of (possibly) layman’s terminology borrowed from economics. Apart from the consistent use of the terminology “capitalistic chemistry” and rephrasing chemical reactions in this way, I will entirely build on earlier published results. The main take-home message will be the application to small metal clusters as models of molecules interacting with an extended surface and what the application of this viewpoint (or equivalently bond preparation^12^) teaches us about the locality of chemical bonding. Here, we will use the concept of “capitalistic chemistry” to remind the reader why the *local* chemical bond of an adsorbate to a metal can be well described by cluster models, while the chemisorption energy requires special consideration.

While this approach is useful to understand the energetics of bond formation in general, I will base the discussion mainly on examples from chemisorption of gas phase molecules with particular emphasis on transition metal surfaces. I will begin with chemisorption from the viewpoint of the molecule, then from that of the metal surface and, finally, conclude by discussing metal clusters as computational models of the extended system. The focus will be on the energetics of the states involved and how they determine the final energy in bond formation. The discussion of the bonding will be in terms of the dominant molecular or atomic orbitals involved, which is applicable also to the solid state as they build up the band structure characteristic of extended periodic systems.^13^

## 2.0 Chemisorption

### 2.1 Chemisorption - The Molecule

**Figure 1:**
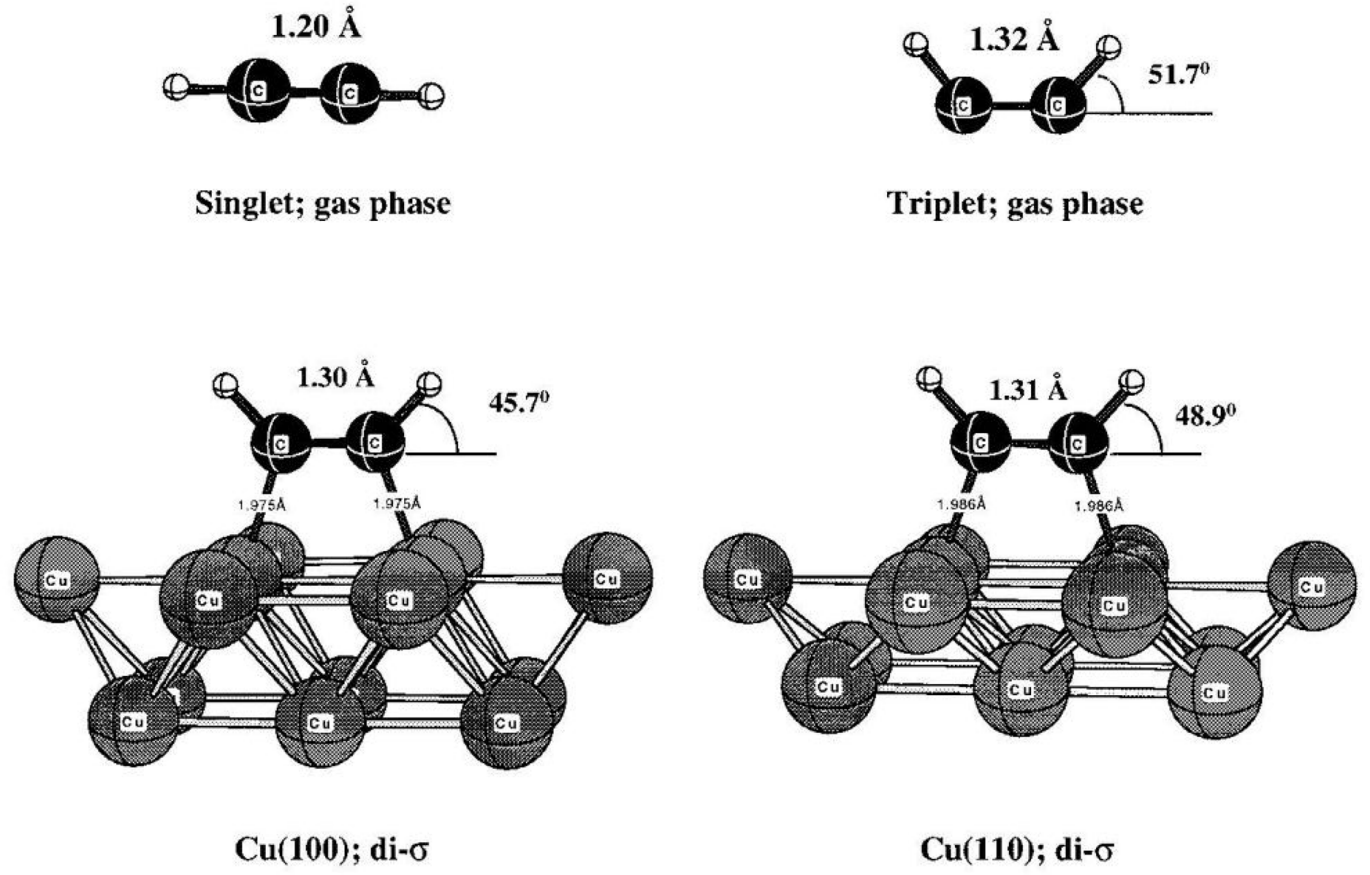
(Top) Acetylene in the gas phase: Singlet ground state (left) and triplet excited state in *cis*-conformation (right). (Bottom) Optimized chemisorbed structure on Cu(100) (left) and Cu(110) (right). Reproduced from ref (4) with permission of AIP publishing copyright 1998.

When acetylene (C_2_H_2_) chemisorbs, the C–C bond length approaches that of a C–C double bond, rather than the short triple bond characterizing the gas-phase molecule (Figure 1). Furthermore, the hydrogens bend up, away from the surface, sometimes confused as a repulsion against the metal. The chemisorption energy is quite small, which in early work seemed to belie the large structural changes. However, the similarity of the resulting chemisorbed geometry to that of the gas phase excited (π → π*) triplet state in *cis*-conformation was early on recognized by Felter and Weinberg^14^ based on vibrational spectroscopy data. It is clear that the closed-shell molecule needs to undergo an excitation in order to form the two σ-bonds to the surface in a lying-down geometry. The bonding is often described in the Dewar–Chatt–Duncanson^15,16^ (DCD) picture of π donation and π* back-donation, but this does not provide information on the energetics involved.

By explicitly taking into account that the molecule, “happy” in its original, gas-phase closed-shell ground state, must invest in a change of both electronic and geometric structure in order to form new bonds to the metal surface, more detailed information can be obtained. By spin-uncoupling^4,17^ one π-bond in the gas phase by exciting an electron into the π* level, two unpaired electrons become available to form new σ-bonds to the surface. Furthermore, this removes one internal π-bond, leading to a C–C bond distance similar to that of the double bond in ethylene and with the hydrogens bending away from linearity. In the *cis*-conformation (Figure 1) the molecule is now prepared to form the required two σ-bonds to the surface for a lying-down geometry. The investment cost to reach this state was estimated by Triguero et al.^4^ as 87 kcal/mol from DFT calculations on the gas phase singlet in its equilibrium structure and the triplet in the geometries extracted from the molecule chemisorbed on various copper clusters. Based on the computed chemisorption energies, together with the investment in the excitation to the triplet state, a C–Cu σ-bond strength of ∼50 kcal/mol was obtained with twice this value clearly giving a net profit (∼13 kcal/mol) and an exothermic reaction, albeit with a rather small net “profit” considering the large “investment” to make this bonding mode possible.

Carter proposed a general approach, quasiempirical valence bond (QVB),^3^ to determine individual bond strengths where one first determines the number and character of the bonds formed, followed by obtaining the experimental or computed heat of formation of the gaseous species in its ground state. The investment to reach the bonding state is then estimated (or computed) for the gas-phase electronic or charge state (and structure) that most resembles that of the chemisorbed molecule. These data can then be used to estimate bond strengths in combination with experimental calorimetry data^7,11^ or to estimate heat of formation if the bond strength is known.^3^ Based on this, Carter and Koel^1^ deduced a value of 53 kcal/mol for a single C–Pt σ-bond and used this value to estimate reactivity in dehydrogenation reactions on Pt(111).^1,2^

Ethylene (C_2_H_4_) provides another example where also a direct, experimental analysis in terms of the DCD model is available.^18^ In terms of “capitalistic chemistry” the relevant state is the triplet electronic state in eclipsed conformation,^4^ which is bond-prepared. This eclipsed triplet ethylene conformation is higher by approximately 19 kcal/mol than the adiabatically excited^3^*B*_1*u*_ state of staggered (twisted) ethylene. However, for the surface the eclipsed conformation is the most appropriate with the two unpaired electrons both pointing toward the surface. The excitation energy to reach this state is again of the order 89 ± 3 kcal/mol,^4^ and this large investment in rehybridization results in a low chemisorption energy (measured as 8 ± 2 kcal/mol on Cu(100) from TPD^19^) in spite of a significant return in individual bond energy of −53 kcal/mol for Cu(110) and 46 kcal/mol for Cu(100) estimated from DFT calculations in ref (4).

The DCD model describes the bonding of unsaturated hydrocarbons to a metal atom or surface in terms of a π donation to the metal and π* back-donation, leading to some empty π character and occupation of the π*. X-ray spectroscopies^20,21^ are ideal to investigate this situation since the photon energy can be tuned to excite from the C 1s level resonantly into unoccupied valence states and thus detect possible depletion of the π state. The thus created 1s core hole has a short (order femtoseconds) lifetime and decays by filling from an occupied valence level with the released energy used to eject an electron (Auger decay) or a photon. The latter is a minority channel for low-Z atoms but forms the basis for X-ray emission spectroscopy^22−24^ (XES) which measures the occupied valence states and can thus detect possible π* occupation. The involvement of the core level makes XES element- and atom-specific, probing the valence levels projected onto the atom with the core hole since an orbital overlap is required for a transition to occur. The 1s core orbital is spherically symmetric, and emission of a photon occurs through a dipole transition, which means that the spectroscopy measures the local 2p character in the valence orbitals, effectively providing an experimental correspondence to a population analysis.^22−24^

The Cu(110) surface is azimuthally ordered with ridges and troughs, leading to directional order of chemisorbed ethylene. Thus, by arranging the crystal parallel or orthogonal to the incoming, linearly polarized X-ray beam, a complete decomposition of the valence orbital p-character could be made^18^ by exploiting the dipole selection rule in absorption. This selection rule requires p-character parallel with the polarization direction of the photon; e.g., excitation into π_*y*_ from C 1s requires a *y*-polarized photon. In emission from the decay of a core-hole state, the direction of propagation of the emitted photon will be orthogonal to the direction of the p-character of the orbital, which can thus be determined by placing the detector accordingly. Furthermore, due to the symmetry of the substrate, the inversion symmetry of the ethylene molecule is closely preserved, providing an additional selection rule; i.e., excitation from an *ungerade* 1s-level can only go into empty *gerade* valence states and *vice versa* for the deexcitation. For the free molecule, this criterion imposes a strict selection rule since only the π* contributes to the lowest excitation, and thus, upon resonant excitation into this state, only *gerade* valence states will be seen in the resulting emission. This is in spite of the energy resolution of the exciting beam being such that the split between the *gerade* and *ungerade* 1s core orbitals cannot be resolved; the selection is due to the symmetries involved. Upon chemisorption the picture changes, in spite of the symmetry being essentially the same. New states appear that result from the bonding to the substrate.

**Figure 2:**
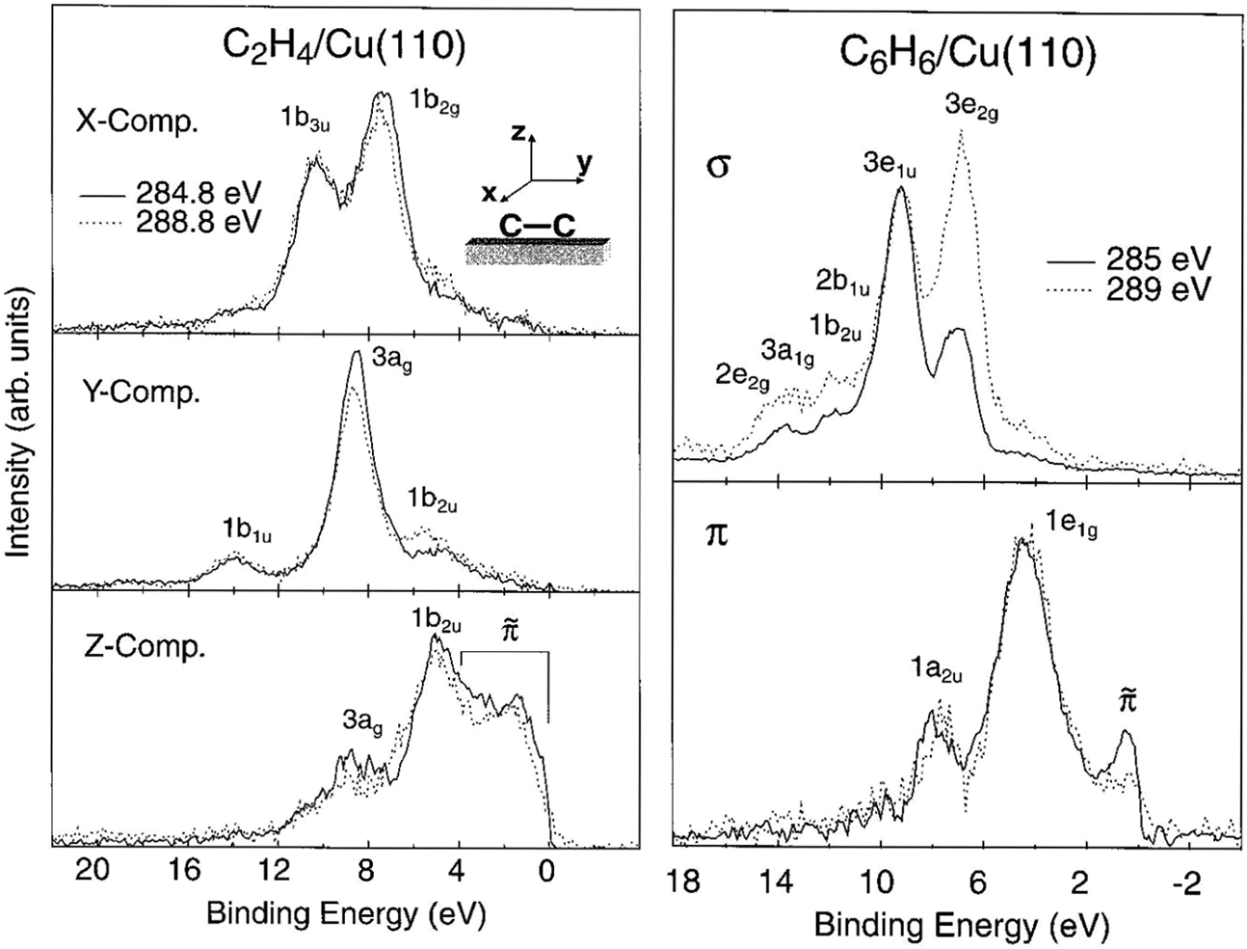
Comparison of symmetry-resolved XES of ethylene (left) and benzene (right) chemisorbed to a Cu(110) surface using two different excitation energies as indicated. The ridges and troughs of the Cu(110) surface extend along the *y*-axis. The spectrum labeled π shows out-of plane components, whereas the spectrum labeled σ shows in-plane components. Reprinted (adapted) with permission from ref (18). Copyright 2000 American Chemical Society.

**Figure 3:**
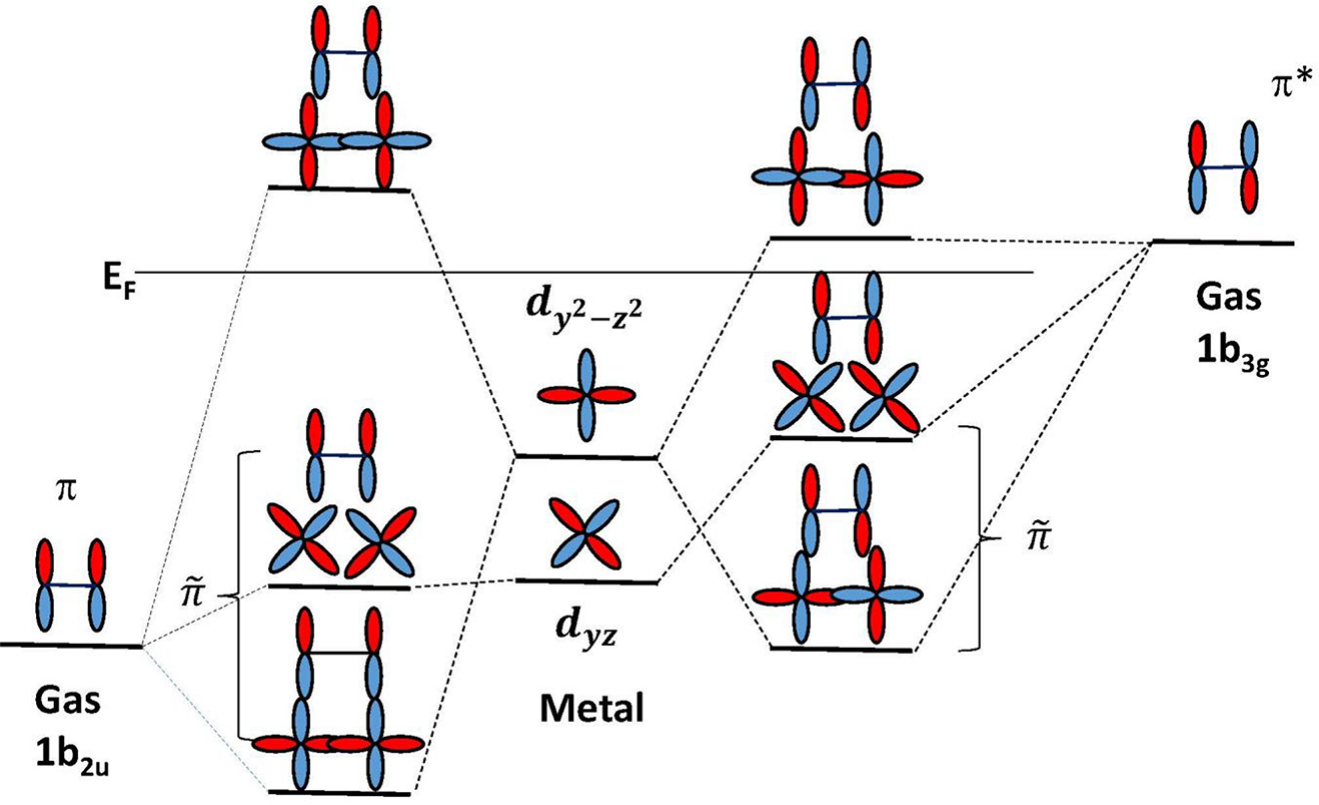
Orbital diagram illustrating bond formation between ethylene and metal d-states involving (left) the 1π (1*b*_2u_) on the ethylene and (right) the 2π* (1*b*_3g_) on the ethylene. The d-state positions indicate their relative positions toward the bottom (left) and top (right) of the d-band. Redrawn based on ref (18). Copyright 2000 American Chemical Society.

Figure 2 shows symmetry-decomposed XES spectra of ethylene on Cu(110) in comparison with the corresponding spectra of benzene on the same substrate. The spectra have been extracted from ref (18) where in both cases two excitation energies were used, with the lower being resonant with the first unoccupied state and the second 4 eV higher. For ethylene, there are rather small differences between the two energies. However, the slight enhancement of intensity from *gerade* states (1*b*_2g_ and 3*a*_g_) in the X- and Y-components indicates somewhat more *gerade* (i.e., π*) character near the Fermi level over *ungerade*, but unlike in the gas phase there is now unoccupied π-character near the Fermi level (1*b*_2u_). The orbitals interacting with the substrate to form the chemisorption bond are illustrated for ethylene in Figure 3. These are seen in the Z-component and indicated by **π̃**—the very similar intensity with the two different excitation energies indicates near-equal contribution of π- and π*-character, thus verifying the DCD^15,16^ picture—as well as that of spin uncoupling^4^ or “capitalistic chemistry” with explicit involvement of the excited triplet electronic state.

In the case of benzene the dependence on excitation energy for the σ-system is similar to that in the gas phase, indicating much less distortion and orbital mixing.^18^ The 3*e*_2g_ state is formally symmetry-forbidden at the lower excitation energy but gains intensity due to vibronic coupling to an antisymmetric C–C stretch mode^25,26^ both in the gas phase and as chemisorbed. In the π-system, the **π̃**-state that appears is enhanced upon resonant excitation and is thus of mainly π*-character indicating some π*-backdonation, while there is no evident π-donation in the spectrum. Thus, for benzene on Cu(110) the orbital interaction with the surface is weak with only minor changes in structure with the hydrogens pointing slightly away from the surface. Here, it is clear that mainly the ground electronic state of benzene is involved in the bonding with minor polarization effects. A more in-depth discussion is provided in ref (18).

Other chemisorption modes for benzene are, however, possible through involvement of the lowest triplet excited states, i.e., the quinoid (boat) and antiquinoid (chair) states which have been proposed in chemisorption of benzene on Ni(100) and Mo(110) based on comparison of computed and measured X-ray absorption (XAS) spectra.^27^ These triplet states correspond to excitations from the highest, 1*e*_1*g*_, orbital into each of the two components of the unoccupied *e*_2*u*_ LUMO orbital. This results in two different *C*_2*v*_ distortions of the molecule, leading to the quinoid (four long and two short bonds) or the antiquinoid structure (four short and two long bonds), both with nearly the same excitation energy (∼90 kcal/mol^4^). The quinoid structure is bond-prepared in the para-position (carbons 1 and 4), while the antiquinoid form has its radical character concentrated on the two longer bonds between carbons 2–3 and 5–6. The quinoid structure was obtained on Pt(111) by allowing the surface to reconstruct in a geometry optimization involving the top four layers of the slab.^28^ This resulted in a lower energy than for the singlet undistorted structure and a chemisorption energy closer to experiment. It should be noted that when a specific excited state is involved this implies a curve crossing, potentially leading to a barrier to access, in the present case, the quinoid state from the gas phase. This was proposed to explain why the quinoid is not observed on Cu(110) in spite of it computationally being the lowest-energy state.^4^

Electrochemical reduction of CO_2_ to C_2_ and C_2+_ products is an important process^29,30^ where the state of CO_2_ bonding to the Cu catalyst has been analyzed by several authors (e.g., refs (31−33)). In the presence of water and enhanced by a negative electrode potential, CO_2_ chemisorbs as an anion with an O–C–O angle around 125°^31,33^ in contrast to the linear gas or aqueous phase geometry. Both the gas phase neutral triplet state and anionic quartet state adopt a similar geometry as found for chemisorbed CO_2_, and both have the possibility to form two bonds to the surface. However, the investment cost in the gas phase for this rehybridization has been estimated^31^ as ∼99 and 114 kcal/mol, respectively, which is too high to be offset by a return of ∼100 kcal/mol from the ∼50 kcal/mol per bond estimated in ref (4). Instead, the anionic CO_2_^–^ doublet state, which in the gas phase has a very similar geometry (133°; *r*_CO_ 1.26 Å) as chemisorbed (124°; *r*_CO_ 1.27 Å), forms the bonding state with a low investment of 13 kcal/mol. The strength of the resulting single C–Cu bond is then consistent with the estimates for acetylene, ethylene, and also OCCO^–^ (from ref (31)). Neglecting the charge state of the adsorbate and only computing the cost to distort the geometry on the gas-phase ground-state potential energy surface leads to a 30 kcal/mol higher cost, which, for the same profit (chemisorption energy), would result in an artificially exaggerated estimate of the bond strength. As outlined in the QVB approach^3^ it is important for consistency to properly identify the state (excited or charged) involved in the bonding.

Acetylene, ethylene, and O_2_ have low-lying π*-states for which an investment in a π–π* excitation can be compensated by the return from bond formation. For a lying-down geometry, with the bond axis parallel to the surface, both heavy atoms need to form bonds to the surface, which becomes possible with a low-lying accessible π*-level. For unsaturated molecules like CO and N_2_, the energy investment to reach the diradical triplet state required for a lying-down geometry is, however, too high to be compensated by the return from formation of two single bonds to the surface. Instead, a partial involvement of the π* is energetically preferable, leading to a polarization of the π toward the metal surface in an upright geometry with formation of a π-bond to the metal. In Figure 4 from ref (20) we show the measured XES spectrum of N_2_ chemisorbed atop Ni(100) in comparison to computed spectra using a Ni_13_ cluster model. By exploiting the core-level shift^34^ between the inner and outer nitrogen atoms the valence orbitals were projected on either the inner or the outer nitrogen. By varying the detection direction, the spectra could furthermore be resolved into σ- and π-contributions.^24,35^

**Figure 4:**
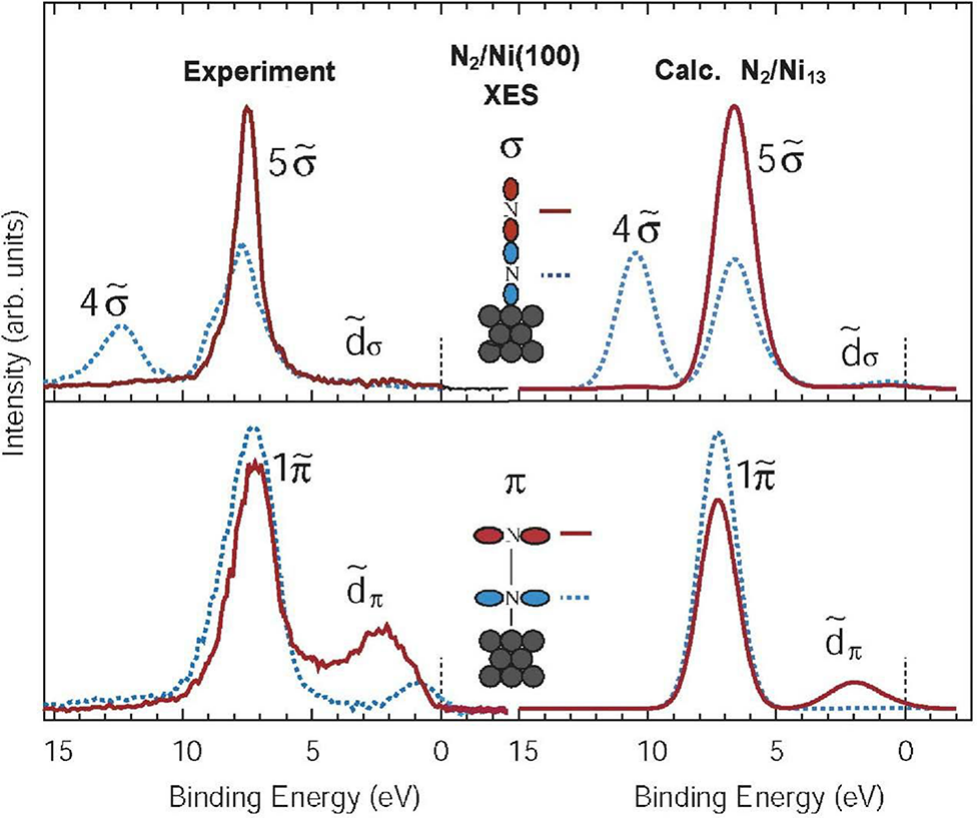
Atom- and symmetry-resolved XES spectra of N_2_ chemisorbed atop Ni(100).^20^ Experiment (Left) in comparison to computed spectra (Right). The top panels show the σ-system and the bottom panels the π-system with valence orbitals projected on either the inner nitrogen atom (blue, dotted) or outer nitrogen (red, full line). Reproduced with permission from ref (20). Copyright 2004 Elsevier Science & Technology Journals; permission conveyed through Copyright Clearance Center, Inc.

In the gas phase, the two nitrogens are equivalent, such that any observed differences upon chemisorption will be due to the interaction with the surface. Thus, the general aspects of an unsaturated π-system interacting with a surface without access to a low-lying π* are more clearly discerned for N_2_ than for the environmentally more highlighted but, in terms of bonding, similar CO. We will return to CO below.

Beginning with the π-system in the experimental XES of N_2_ on Ni(100),^24,35^ we observe a higher intensity for the inner nitrogen in the main π-peak at ∼7 eV binding energy (marked as **1π̃** to highlight changes compared to the gas phase), showing that the molecular π-orbital polarizes *toward* the metal. At lower binding energy there are new states, absent in the gas phase, where at 2–3 eV only the outer nitrogen contributes, forming a lone-pair **d̃_*π*_** state,^20^ while close to the Fermi level both atoms contribute more equally, as in the molecular π*. The experimental spectrum can now be directly seen as an allylic orbital interaction^35,36^ where Ni d_π_ and N_2_ π and π* form three new orbitals: totally bonding **1π̃**, a nonbonding **d̃_*π*_** (no contribution on the inner nitrogen), and a totally antibonding, π*-like orbital on the molecule, forming an allylic orbital diagram. The relative contributions from N_2_ and the metal are furthermore reflected by the intensity in the different regions of the spectrum. There are more contributions from nitrogen to the **1π̃** peak and more metal character in the **d̃_*π*_** region; with the core hole on one of the nitrogen atoms, only contributions from the nitrogens are seen, and the metal character is inferred by the loss of intensity.

The observed orbital changes are in agreement with the traditional Blyholder picture^37^ for the π-system but not with a simple frontier orbital picture of π* back-donation; the orbitals change, and new ones appear.

For the σ-system, the XES measurement shows a different picture from the traditional σ-donation. The spatially more extended 5σ polarizes *away* from the surface, while the 2p character of the 4σ is only seen on the inner nitrogen. Here, the antibonding σ* orbital is very high in energy which results in less flexibility to modify the orbitals in the σ-system, making the σ more stiff, resulting in repulsion^38^ that needs to be alleviated to allow for the π-bond to form.

**Figure 5:**
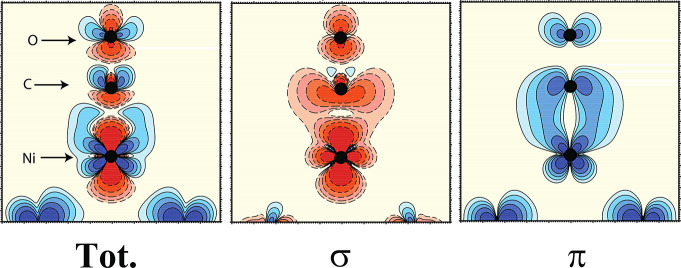
Charge density difference plots of CO on a Ni_13_ cluster showing charge redistribution upon chemisorption atop the central Ni atom in the cluster. (Left) The total difference taken as chemisorbed minus the gas phase molecule and the cluster. The center and right panels show the changes in density resolved into σ- and π-contributions showing the loss (red) in σ for both the molecule and the d_σ_ of the metal and gain (blue) in the overall π-system with the formation of a π-bond to the metal. Reproduced from ref (5) with permission of Springer Nature BV copyright 1994; permission conveyed through Copyright Clearance Center, Inc.

In Figure 5 we show computed density difference plots for the similar system CO chemisorbed on a 13-atom cluster model of the Ni(100) surface.^5^ CO binds similarly to N_2_ but with molecular orbitals polarized already in the gas phase, which makes N_2_ a simpler system to analyze.

The plots were generated as the difference in density between the chemisorbed system and the sum of the gas-phase CO and cluster. The take-home message here is seen from the symmetry-resolved plots where it is clear that the entire σ-system loses density and also the metal atom that CO binds to. In particular, the metal d_σ_ density clearly is reduced due to the interaction with the closed-shell 5σ. On the other hand, the entire π-system gains density, which on the metal atom corresponds to a rehybridization among the 3d orbitals to reduce the repulsion and gain the bonding to the adsorbate, i.e., a d_σ_ to d_π_ excitation.

Depending on the metal, substrate structure, coverage, temperature, and influence from coadsorbate species, CO can adsorb in different sites. These often coexisting phases indicate only small energetic differences for different sites, which has been interpreted as an indication of rather similar bonding. However, vibrational spectroscopy clearly shows that the CO intramolecular bond is affected differently in the different sites with the CO stretch frequency decreasing with increasing coordination to the metal substrate.^37^ This indicates a weakening of the internal CO bond and has in the past been discussed in terms of backdonation from the metal to the unoccupied antibonding CO 2π* orbital.^39−42^ However, direct experimental measurement shows a different picture.

On Ni(100) and H/Ni(100), CO can occupy atop, bridge, and hollow positions with relatively minor differences in chemisorption energy but with very big differences in electronic structure as measured by Nilsson and co-workers^43^ using XES. A similar allylic orbital diagram as for N_2_ is applicable also for CO chemisorption with the metal d_π_ and CO 1π and 2π* forming totally bonding, nonbonding, and totally antibonding combinations. In the gas phase the occupied 1π is polarized toward the more electronegative oxygen, and consequently the 2π* is polarized toward the carbon. CO binds to the metal with the carbon end, and already in the atop position the carbon contribution to the **π̃** orbital forming the bond to the surface has increased compared to gas phase CO. The carbon contribution to this π-bond increases further for bridge-adsorbed CO, and when adsorbed in hollow position, carbon and oxygen contribute close to equally to this orbital.^43^ The intensity in the chemisorption-induced lone-pair-like **d̃_π_** state follows the same trend, becoming strongest for CO in the hollow position. The polarization in the π-system is always *toward* the surface and increases with the number of interactions with substrate atoms; the interaction is attractive.

The σ-system follows a similar trend of greater changes with increasing coordination but with polarization *away* from the metal; to obtain the return from the π-bonding, the molecule has to move close enough for a π-bond to develop, which comes at the cost of σ-repulsion. Clearly, there is a balance between the enhanced π-bonding and the increased repulsion in the σ-system with increasing coordination.

An estimate of the repulsive σ interaction has been given^43^ by calculating the initial repulsion between the unperturbed CO molecule and the substrate at the equilibrium bonding distance. The computed initial Pauli repulsion was obtained as 3.3, 5.6, and 10.3 eV for on-top, bridge, and hollow sites, respectively, which directly scales with the amount of σ-polarization seen experimentally in the measured XES spectra.^43^ In spite of this very large difference in initial investment in terms of changes to the electronic structure, the resulting chemisorption energies (profits) are quite similar and more than one type of site can be occupied simultaneously. It is clear that the changes in electronic structure provide a better indicator of the character of the bonding (chemisorbed, physisorbed, etc.) than the resulting chemisorption energy. It is also clear that, with such large variations in investment cost and return for chemisorption in different sites with similar net resulting chemisorption energy (profit), it becomes very challenging to obtain the proper balance between these effects in computations and thus to correctly predict the preferred adsorption site. The classical example is CO/Pt(111),^44,45^ where the delicate balance between σ-repulsion and π-bonding at different sites in computations has led to incorrect site assignments from theory.^44−46^

We end this section by commenting on the d-band model of Hammer and Nørskov^47−50^ that relates the chemisorption energy for an adsorbate on a transition-metal surface to the position of the d-band. Since all transition metals have a half-filled, broad conduction (s-) band, the interaction of this band with an adsorbate will be similar for all metals and result in a broadening of the adsorbate state; the differences lie in the energy position and occupancy of the more narrow d-band.^47−50^ For an atomic adsorbate the latter interaction results in the formation of bonding and antibonding states, which are separated in energy. The resulting chemisorption energy depends on the relative occupancy of bonding and antibonding states, which in turn depends on the occupancy of the relevant atomic d-shell or, equivalently, the position of the d-band relative to the Fermi level. For a given split between bonding and antibonding states a lower d-band position “drags” antibonding states down below the Fermi level, while a higher d-band “pushes” them above, i.e., leaving them unoccupied and thus strengthening the bonding; as a consequence binding energies increase going from the right to the left along a transition row. This picture has been verified experimentally in a comparison of atomic nitrogen on Cu(100) and Ni(100) using X-ray absorption spectroscopy (XAS) to probe the occupation of the antibonding state and XES for the bonding state.^51^

For CO and N_2_ the bonding interaction instead involves three levels (metal d_π_ and the π and π* of the molecule), forming three new states. The split between the molecular π and π* is fixed and independent of the metal, while the d-band position varies and determines the relative occupancy. As a consequence, the resulting chemisorption energy increases going to the left along a row (combined with the σ-repulsion becoming smaller due to the reduced d-occupancy); again, a higher-lying d-band results in less occupancy of the antibonding state and thus a stronger bond.

Even though the involvement of the excited π* state is only partial, the amount and energy cost of this involvement relative to the gas-phase ground state can in principle be determined using a cluster model. Computing the, e.g., CO ground state and excited π* state for the gas-phase molecule in the chemisorbed geometry gives the cost to change the geometry in the ground-state electronic structure and the excitation energy in this geometry. These normalized and orthogonal wave functions can then be used to project out the corresponding character in the interacting chemisorbed molecule and an estimate of the cost of this contribution to the rehybridization obtained, based on the fraction of each component and their energies. This only gives the π-symmetry but can be used to investigate trends, e.g., along a transition row.

### 2.2 Chemisorption - The Metal

The necessity to include changes in the substrate and not only the adsorbate when analyzing the balance between investment and return to assign a bond strength has been pointed out by Gross et al.,^7^ where for ethylene on Pt(110) it was argued that the cost to restructure the surface was small enough to be neglected, while the experimentally measured reconstruction energy for Pt(100) hex to (1 × 1) is too large to ignore. The “added row” reconstruction^52,53^ of Cu(110) upon adsorption of oxygen is another classical example where the changes in the substrate need consideration, e.g., by computing the energy difference between the initial and reconstructed surface without adsorbate.^54^ Another situation is adsorption-induced segregation as seen, e.g., in adsorption of oxygen on a thin film of Pt on Cu(111)^55^ where Cu atoms segregate to the surface due to the stronger binding of oxygen to Cu than to Pt. The flexibility and reconstruction of surfaces upon interaction with adsorbates and under reaction conditions have been discussed extensively, e.g., by Somorjai and co-workers.^56,57^ Here, we will focus on electronic structure changes in the substrate atoms.

We note that, particularly for metals, the density of states around the Fermi level and the number of neighbors that can participate in charge transfer make it often impracticable to determine what excited states are involved to modify the bonding and structure in the metal. Determining the energetics in such cases is less straightforward and requires a decomposition of the energy contributions, which can be done using various energy decomposition analysis (EDA) schemes.^58−65^ For local bond formation, as the π-bond when CO or N_2_ chemisorbs, it is, however, still relevant to consider the change in metal electronic state, as exemplified by the d_σ_ to d_π_ excitation to enhance the bonding. However, this is now embedded in a density of states, electronic and vibronic, that are available to reduce the cost to prepare for the local bond formation. Here is a strength of EDA with its emphasis on the energetic contributions from various physical interactions^62,63^ or classes of orbitals,^64^ rather than from specific orbital interactions when such cannot easily be identified.

Before discussing water on Pt(111) as a specific example of the interplay between electronic structure changes and charge transfer in the metal it is important to note the general view to excited states that is applied here. The basis is that all changes in the electronic structure compared to an initial situation must occur through involving states that in the initial situation were unoccupied and thus formally excited. The associated excitation energies depend on the level spacing, which for a molecule in the gas phase is discrete with large steps for the first few valence excitations, while in the condensed phase the lowest electronic excitation is determined by the band gap (or lack thereof). Energy and momentum are always conserved, which means that for an exothermic reaction to occur the excess energy has to be dissipated for the product to be stabilized; otherwise, there is only an attempt at bond formation, and the reactants separate (see discussion of CO oxidation below). Typically, phonon modes in the metal are excited (heat), but the excess energy can also be stored as potential energy through reconstructing the surface or through charge density rearrangements in the substrate that would be of higher energy in the absence of the adsorbate. The changes in the metal charge density in the presence of the hydrophobic first layer of water on Pt(111) are an example of the latter.

Pt(111) is a flat surface to which a single water molecule binds through a σ lone pair with a resulting chemisorption energy (0.1–0.4 eV) comparable to the formation of a water–water hydrogen bond; the dipole/image dipole attraction is too weak at the typical water metal distances to contribute significantly.^66^ In the first monolayer, on the other hand, the molecules alternate between contacting the metal through an oxygen lone pair and through an OH (H-down); the rehybridization of the waters in the two different bonding modes has been measured separately using XES.^67^

The lone-pair σ-interaction with the metal is often discussed in terms of forming a dative bond where mainly sp density is polarized away (“digging the s-band hole”^68^) and the doubly occupied lone pair is stabilized through electrostatic interaction with the resulting positive ion core. However, for CO and N_2_ it was pointed out early on that the lone-pair σ-contribution is repulsive and that the main bonding contribution is due to the π-system.^38^

The σ-interaction for CO and N_2_ becomes repulsive due to the possibility to form bonds in the π-system, which requires a shorter distance for sufficient overlap causing repulsion of the 5σ against the d-states. Since the main repulsion with transition metals is against the d-states, the magnitude will depend on the d-occupation with decreasing repulsion for metals with less filled d-states.^69^ However, for water adsorbed on metals, the σ-interaction instead becomes attractive. On Pt(111) the molecules in the first water layer alternate between lying parallel to the surface (binding through oxygen) and having one hydrogen pointing to the metal (binding through hydrogen).^67^ The binding through oxygen occurs through a dative bond involving the oxygen lone pair contacting the metal ion core after sp-charge density has been polarized away.^68,67^ Here, a typical O–Pt distance is around 2.35 Å compared to ∼1.75 Å for O–Pt when CO binds to Pt(111), and there is thus significantly less overlap with the d-states on the metal; however, the interaction still requires a rehybridization through a d_σ_ to d_π_ excitation on the metal and loss in the oxygen lone-pair density pointing toward the metal.^67^

**Figure 6:**
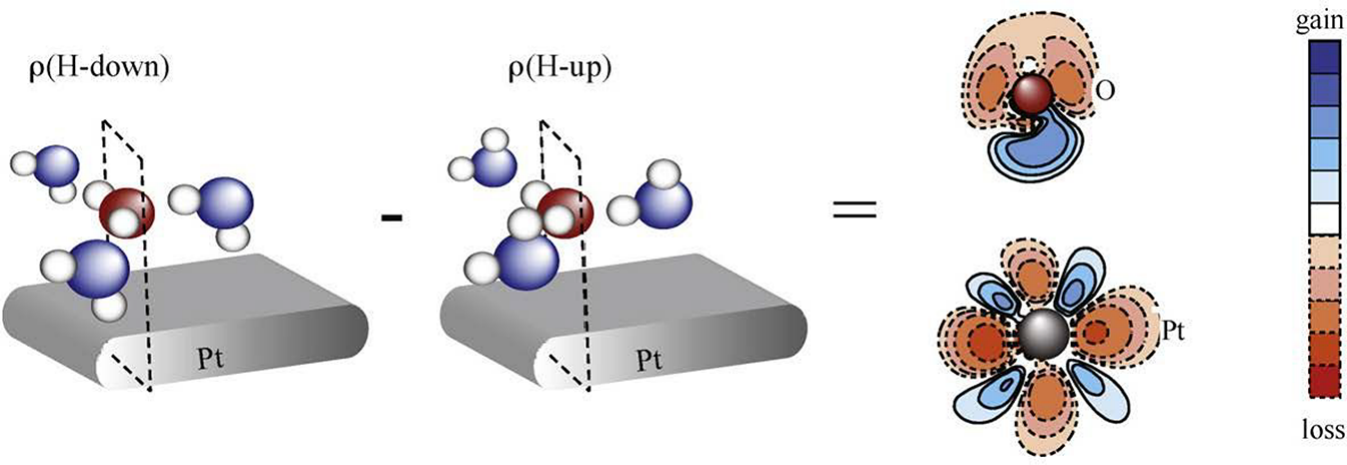
Hydrogen switch. Density difference between H-down and H-up configurations of water on Pt(111). The cut is through the central oxygen and the underlying Pt. Reproduced from ref (66) with permission of Elsevier Science & Technology Journals copyright 2010; permission conveyed through Copyright Clearance Center, Inc.

At cryogenic temperatures the first layer of water on Pt(111) is hydrophobic toward attachment of subsequent layers.^70,71^ The origin of this effect was investigated in detail by Schiros et al.^66^ by calculating the second-layer H-bond strength to the oxygen in a lying-down geometry (red molecule in Figure 6) in the absence of the surface and finding 0.14 eV, which was reduced to 0.02 eV when the first layer was put in contact with the surface; the H-bond interaction with the oxygen lone pair was essentially eliminated.

In Figure 6 we show the charge-density difference between the H-down and H-up situations for water on Pt(111) in a cut including the oxygen lone pair and the contacted Pt atom. In the H-down (binding through hydrogen) situation the O–H σ* is available to accept charge from the Pt which allows the Pt contacted by the oxygen to transfer some charge to its neighbor to further reduce repulsion and thus increase the return from bond formation; flipping the H-down molecule to H-up disrupts this channel, and the circuit of charge transfers is no longer closed.^66^ Thus, here there is charge transfer within the metal that is facilitated by the particular interactions in the surface adsorbate layer.

In the figure, it is clearly seen that the H-down situation leads to a polarization of the oxygen lone pair toward the Pt (relative to H-up) and facilitates the d_σ_ to d_π_ excitation. The depletion of the outward-pointing component of the oxygen lone pair then leads to the significantly weakened attachment energy for the next layer. However, these electronic structure effects are rather subtle and require careful EDA in order to set up an approximate energy budget. It should, however, be noted that at ambient temperatures there is sufficient disorder that subsequent layers actually do wet and form a continuous liquid film,^72^ albeit with significant fluctuations of importance in modeling electrochemistry.^73^

The importance of the Pt d_σ_ to d_π_ excitation in reducing the σ-repulsion to allow the formation of the dative bond with the oxygen lone pair is evidenced experimentally by comparing to isostructural Cu(111), which is hydrophobic (i.e., water does not bind) both at cryogenic and ambient conditions.^74^ Here, the sp-density is similar to Pt(111), so the electrostatic contributions are comparable, but the closed-shell d^10^ occupation of copper leaves no room for rehybridization within the 3d shell; investment in an excitation from the top of the Cu d-band to the Fermi-level is too costly to be offset by the strength of a dative bond. However, adding 0.12 ML atomic oxygen to Cu(111) leads to OH^–^ groups that bind strongly electrostatically to the surface and act as anchor points for water through strong water-to–OH^–^ hydrogen-bonding.^74^ Since the d_σ_ to d_π_ excitation is too costly an investment in Cu, wetting of Cu(111) thus occurs only through the presence of a stabilizing group, e.g., OH^–^.

### 2.3 Chemisorption - Clusters to Model the Extended Surface

The cluster is in reality a small molecule with discrete energy levels where the electronic structure typically depends on the number of atoms in the cluster and may or may not include a sizable band gap. For, e.g., alkali metal and copper clusters this leads to the well-known even–odd alternations in reactivity and other properties as the clusters alternate between closed- and open-shell electronic structure with the addition of more atoms. The discreteness of states and number-dependent electronic structure are direct and real physical effects due to the confinement of the electrons in a finite volume of space (particle in a box). If used as models of the extended surface this leads to slow and erratic convergence of the computed chemisorption energy with cluster size—if computed as the difference between the ground state of the cluster and that of the chemisorbed system.

However, in order for the bond between the surface and adsorbate to form, the electronic structure of both surface and adsorbate must change, which can only be achieved through mixing with excited states. For the extended metal surface, such excitations occur around the Fermi level at zero or small energy cost. For the metal cluster, however, a finite excitation energy may or may not have to be invested to reach the bonding state, and this rehybridization cost reduces the resulting computed chemisorption energy, i.e., the profit. This investment needs to be taken into account to obtain chemisorption energies from a cluster model that better reflect the properties of the extended metal.

By calculating this excitation energy for the cluster and adding it to the computed chemisorption energy, surprisingly accurate binding energies are obtained also from quite small cluster models.^12,75−78^ Thus, the *local bond* is well-defined already for small clusters. However, since the *chemisorption energy* includes the investment cost to excite the cluster to the bonding state and this investment in rehybridization varies strongly with cluster size and shape, the result is the observed strong variations, if this is not accounted for.

Siegbahn and co-workers investigated the convergence of chemisorption energies with cluster size for a number of systems^12,75−78^ and determined sets of rules to in effect take into account or minimize the investment cost to reach the bond-prepared state for a cluster. In the case of, e.g., hydrogen adsorption a matching open-shell *a*_1_ orbital is required on the cluster to pair with the 1s of the hydrogen to form the covalent bond.^75^ Lacking this, i.e., if the states in *a*_1_ symmetry are doubly occupied, then an electron has to be promoted to an excited level to allow the bond to form. The same covalent bond is formed, with similar bond strength, but now with the profit from bond formation reduced by the investment to reach the bond-prepared state. If this is not taken into account, then the computed chemisorption energies show strong variations due to the varying investment costs.

A similar result was obtained for methyl bonding to various cluster models of the Ni(111) surface.^78^ In this case the bonding is through the unpaired 2p_*z*_ electron, and the requirement is again an open-shell *a*_1_ orbital on the cluster for the bond to form without investment in an excitation on the cluster. Atomic fluorine has one open shell, and similar to hydrogen and methyl, the optimal binding is for both fluorine and the cluster to have an unpaired *a*_1_ electron (p_*z*_) for a highly polarized bond that “fits into” the electronic structure of the cluster.^76^ The high electron affinity of fluorine makes chemisorbed fluorine on both Cu(100)^79^ and Ni(100)^76^ nearly completely ionic, and it might be expected that the ionization potential of the clusters would affect the resulting chemisorption energy. However, no correlation between the computed chemisorption energies and the ionization potential of the various clusters could be found.^76^

The chemisorption energy for oxygen was, on the other hand, found to be less sensitive to cluster size effects.^77^ Atomic oxygen has three identified bonding modes when interacting with the metal,^77^ which allows more ways to minimize the investment cost, as demonstrated for Ni(100) and Ni(111), and thus making extracting reliable chemisorption energies less challenging;^77^ between different cluster models there are smaller variations in investment cost that affect the profit from bond making (i.e., chemisorption energy). Given a fixed return in terms of bond strength it is good business to minimize the investment.

Bond formation between an adsorbate and atoms of the substrate requires an overlap between the electronic states, which scales approximately exponentially with their spatial separation. This makes the contribution of covalency quite local, and even for highly ionic bonding, the short screening length of metals allows for a reliable estimate of the return from bond formation once the investment cost has been taken into account.^76^ This conclusion might seem at odds with a band-structure picture, but by taking combinations of the occupied bonding band states these can be localized to maximize the contributions from individual bonds. The success of the d-band model^47−50^ and associated scaling relations^80−84^ largely depends on the locality of bonding where for the adsorbate the position and split between bonding and antibonding states is fixed, while the position of the involved states on the metal varies along a row of the periodic table and thus determines the mixing with the states of the adsorbate.^5^ The local character of adsorbate bonding has furthermore been noted by Campbell and co-workers.^8^

A distinct advantage of representing the extended system using periodic boundary conditions is the inclusion of coverage effects and additional active or passive species in a reaction sequence, as well as effects of surface restructuring due to chemisorption or reaction conditions. However, accounting for these contribtions typically precludes applying higher-level quantum chemical techniques, e.g., CCSD(T), RASPT2,^85^ diffusion Monte Carlo, or higher-order Density Functional Theory (DFT),^86,87^ due to the increased computational cost with system size. The high-level techniques can, on the other hand, easily be applied to smaller cluster models of the system. Based on the local nature of the chemical bonds one may thus attempt to combine periodic and cluster calculations to leverage the strengths of both approaches. Such a strategy was recently developed and employed by Alessio et al.^88^ who combined DFT calculations on the periodic system with a correction for the local bond strength from CCSD(T) calculations on smaller cluster models to determine a state-of-the-art theoretical adsorption energy of CO on MgO(001). Small molecules interacting with an undercoordinated Ti site in TiO_2_(110) were studied by Neese and co-workers^89^ using a combination of periodic boundary calculations at the rPBE-D3 level and DLPNO–CCSD(T) calculations on embedded cluster models. Other applications to oxide systems have been reviewed by Sauer.^90^ For the case of a transition metal, Hu et al.^91^ have shown that using hybrid functional B3LYP on smaller clusters as a correction to the local bonding correctly predicts the site where CO adsorbs on Cu(111). This approach has recently been extended to open d-shell metals for a number of adsorbates by Araujo et al.^28^ using the M06^92^ hybrid functional as correction of the local bond strength.

Since the correction is based on the difference in the computed interaction energies for the adsorbate and cluster in the same geometry and electronic state between the DFT model and the higher-level approach, the correction becomes largely independent of the size of the cluster. Furthermore, by retaining the optimized geometry from the periodic DFT calculation, the computed energy difference between DFT and a higher-level approach becomes a correction to the interaction energy in the geometry relevant for the periodic calculation; this correction is then added to the interaction energy from the periodic calculation. The combination of more accurate calculations on the cluster model and the advantages of the periodic calculation to include effects of surface relaxation, band structure, coverage and coadsorbates, as well as localizing transition states, holds the potential to provide accurate and reliable energetics that are largely independent of the choice of functional in the periodic calculation. For the 38 included covalent and noncovalent chemisorption or reaction energies, mean absolute errors compared to experiment of 2.2 kcal mol^–1^ and 2.7 kcal mol^–1^, respectively, were obtained by Araujo et al.^28^ We note, finally, that, since the aim is only to correct the local bond using the cluster calculations, it is sufficient to ascertain that the same electronic state of the cluster is used both in the higher-level and simpler DFT calculations on the cluster. The connection to “capitalistic chemistry” is then simply the realization^12,75−78^ that, already for rather small clusters, the bond strength can be well-described; the large variations in computed “profit” largely disappear if the variations in investment in terms of rehybridization or bond preparation^12^ are taken into account.

Regarding the cluster with adsorbate as a small molecule to which we apply two different quantum chemical techniques, one more approximate that is also suitable for the periodic system and a more accurate technique that is restricted to the smaller system, it seems clear that the local interaction with the cluster will be more reliably described by the higher-level approach. This thus provides a general basis for correcting the local component of the interaction obtained with the more approximate technique in the periodic calculation.

With this viewpoint, i.e., that in any given geometry a higher-level (correctly balanced between gas phase and chemisorbed) approach will give a more accurate result for the interaction between a molecule and cluster, the same philosophy should be applicable also to transition states and barriers. That is, one would determine the transition state (TS) in the periodic calculation and then extract a cluster large enough to support the reactants and correct the interaction energy using the higher-level technique. This was done by Araujo et al.^28^ for a small set of five barriers and comparing with the experimental results from the SBH10 database^93^ with a resulting reported mean absolute error of 1.1 kcal mol^–1^. The computed barriers were corrected for the difference in zero-point energy between reactants and the TS, and the surface was furthermore allowed to relax at the TS geometry in the periodic calculation to determine the geometry, as was the case in the simulations to derive the SBH10 database.^93^ In the later SBH17 database^94^ it was argued that the surface atoms would not have time to relax during the passage through the TS in the studied molecular beam experiments and that zero-point corrections should not be made. Based on this reasoning, the work of Araujo et al.^28^ was strongly criticized.^95,96^ However, the importance of the zero-point energy variations in a reaction seems well established, while the time scale of passing through a TS may not be.

The lowest-energy state of a molecule or adsorbate is the lowest electronic energy plus the zero-point energy, which both will differ between the gas phase, chemisorbed, and TS and are usually included when computing barriers. The time the system spends at the TS will depend on the available kinetic energy along the reaction coordinate. In a classical conservative system kinetic energy is converted to potential energy when approaching the turning points in a bound system and is zero at the minimum initial kinetic energy required to cross over the barrier. This effective slow down of a reaction has been observed experimentally.^97^

In a recent study^97^ of CO oxidation on Ru(0001) the reaction was initiated by an optical femtosecond pulse on the CO/O/Ru(0001) coadsorbate system and followed using femtosecond X-ray pulses from a free-electron X-ray laser. After a delay of ∼1 ps a clear signal of molecules at or near the TS was obtained. This was evidenced by the appearance of intensity in the X-ray absorption spectrum in the σ* region; this corresponds to the antibonding component of a molecular σ-bond, and its position at relatively low energy indicated a longer bond than the final C–O bond in CO_2_.^97^ It was argued that the initial step in the formation of the new bond was seen and that this was possible since, contrary to common belief, the reaction *slows down* at the TS where the potential energy is the highest and the kinetic energy is at its lowest. Quantum mechanically this situation corresponds to the vibrationally highly excited oxygen atoms spending most of the time at the classical turning points in the vibrational potential of the surface-confined system, which for a system with energy close to the barrier height means in the vicinity of the TS geometry. The low yield (∼10%) of CO_2_ products was taken as indication that most attempts failed, and the reactants separated back without resulting in products. Essential to make these observations was the simultaneous initiation of the reaction with the optical laser such that a large enough population synchronously arrived in the vicinity of the TS; under steady-state conditions the number of molecules undergoing reaction at any given time is typically too small to allow those specific molecules to be detected.

In a molecular beam experiment, the impinging wave packet will undergo interference that builds up probability in the region of the barrier with extent depending on the kinetic energy. To which degree this affects the surface will depend on how large this probability is and also on the properties of the surface. Present day computational DFT techniques applied to sufficiently thick and extended slab models can reliably obtain the zero-Kelvin optimized structure of the surface with the reactants at the TS. However, this should then likely be weighted with the probability of finding the system in this region or equivalently taking into account the time spent in the vicinity of the TS. Whether or not the surface has time to relax at the TS thus seems not completely established and could possibly be addressed in fully quantum reactive scattering simulations, allowing also the surface atoms to relax and preferably at the relevant temperature. This, however, represents a great challenge at the present time.

## 3 Concluding Remarks

In the present contribution, I have emphasized the importance of considering the full investment in terms of rehybridization cost that a reacting molecule has to make in order to arrive at the proper electronic and geometric state for bonding. The return from this investment, i.e., the total bond strength, minus this investment gives the exothermicity of the reaction or, in business terms, the profit. The rehybridization cost or investment can be obtained for a molecule by determining the gas-phase electronic (and/or charge) state that best represents the situation in the final bonded state. This can be found either from the experimental spectrum of the gas phase molecule or by computing the energy difference between the ground and excited state. Adding the measured or computed chemisorption energy (in heterogeneous catalysis) or exothermicity (in the general case) gives the total strength of the bonds that are formed.

Knowing the bond strength (return) and rehybridization cost (investment) allows predicting whether a specific bonding mode will be favorable. A typical situation is found for unsaturated π-systems where a π to π* excitation results in two unpaired electrons that can form two σ-bonds if their bond strength together is sufficient to yield a profit after this investment. For acetylene and ethylene this is the case, and they are typically bound with both carbons bonding to the surface when chemisorbed. For CO and N_2_ this excitation is too costly, and instead they typically bond to the surface through the π-system in a standing-up vertical geometry. Rather than the frontier-orbital picture of σ-donation and π*-back donation in CO or N_2_ binding to a surface, direct measurement of the orbital character using X-ray emission spectroscopy^35,43,69,98,99^ shows that all orbitals change and new ones are formed. The metal d_π_ together with the molecular π and π* form an allylic orbital diagram with bonding, nonbonding, and antibonding states; the π bond is seen to increasingly polarize toward the surface with increasing coordination, i.e., from top to bridge to hollow. At the distance suitable for π-bonding, the σ-interaction becomes repulsive,^38^ and to alleviate this, the molecule (and the metal atom) has to invest in charge transfer from the σ-system to the π; for the metal this corresponds to a d_σ_ to d_π_ excitation. In this case, as also in the general case of electronic structure rearrangements in the metal surface, the rehybridization is achieved through partial mixing with excited states with degree of mixing dependent on the position of the involved state on the metal relative to the states on the adsorbate. Since the adsorbate states are fixed in energy, the relative bonding contributions from metal and adsorbate, as well as repulsion, will depend on the occupation on the metal such that trends along the transition rows can be determined based on descriptors developed for the metals.^48,50,80−84^ However, working out the budget when excited states are only partially involved requires more advanced energy decomposition computational techniques.^58−65^

The main message that carries the potential to lead to enhanced accuracy regards using smaller cluster models, carved out from the standard periodic-boundary DFT model, to improve the accuracy through correcting the local bond strength. This entails the realization that the often-observed strong variations in computed chemisorption energy with cluster size and shape can be essentially eliminated when the variations in investment to reach the bonding state of the cluster are taken into account. In particular, this indicates that the chemisorption bond is quite local, which suggests correcting the computed energetics from the periodic calculations by applying more advanced (costlier but doable) techniques to the smaller cluster system.^28,88−91,100^ The basis for this is that any system in the same geometry and electronic structure will be better described at a higher level of quantum chemical approximation or higher rungs on Jacob’s ladder of DFT^86,87^ (assuming a balanced description of the gas phase and adsorbed system in chemisorption). Formally, this should apply independent of geometry, i.e., for both equilibrium and transition state geometries.

An interesting question in connection with the transition state optimization regards whether the surface has “time to relax”, i.e., whether surface relaxation should or should not be included when computationally determining barriers.^94−96^ In recent X-ray free-electron laser measurements of CO oxidation on Ru(0001), with CO coadsorbed with oxygen, a clear signal of molecules at or in the vicinity of the transition state was observed.^97^ This was possible through the time resolution of the X-ray laser and through initiating the reaction synchronously to generate a large enough population at the transition state. It was argued that, due to conservation of energy, the reaction should slow down at the top of the barrier as kinetic energy is converted to potential. Whether this slow-down is sufficient for the surface to respond to the reaction, i.e., if surface relaxation should be included in the energy balance for the barrier, is not clear from these data however.

Additional benchmarks for theory have recently become available through *operando* X-ray photoelectron spectroscopy (XPS) that could provide further information on the relevant energetics in reactions. This technique has recently been extended by the group of Nilsson^101,102^ from traditionally low pressures to pressures around 1 bar which are closer to realistic reaction conditions. The measurements at different pressures and temperatures of the state of the surface in terms of species and coverages under relevant reaction conditions provide detailed information beyond the yield of products to which results from, e.g., microkinetic modeling (MKM), can be calibrated.

MKM relies on computed chemisorption energies and barriers and is expected to describe the catalytic yields and intermediates, assuming that the surface in the experiment is as assumed in the model, all relevant reactions are included, and the energetics are computed accurately enough. The high-pressure XPS setup has been applied to study a large number of reactions, including CO hydrogenation on Ni,^103^ CO and CO_2_ hydrogenation on stepped Rh(211),^104^ the state of zinc in methanol synthesis,^105^ the Haber–Bosch process,^106^ and carbide formation in Fischer–Tropsch synthesis on Fe(110).^107^ Coverages of carbon and oxygen containing species during CO hydrogenation on Rh(111) at 150 mbar^108^ were recently modeled by Valter-Lithander et al.^109^ using computed data for the assumed reaction steps. The modeling was coupled to a genetic algorithm (GA) that acted to modify the computed values to obtain agreement with the measured coverages and XPS spectra at each temperature. The intended philosophy was that values that the GA modified beyond the assumed uncertainty could be independently calibrated through combining periodic and cluster calculations and fixed at the verified value. Failure to reproduce the experimental coverage data would then indicate missing components in the model (side reactions, coadsorbates, steps, adatoms, etc.). The GA is a very powerful tool to explore the reaction parameter space and the assumed MKM model. If the computed reaction scheme and energetics are close to correct, including barriers with or without relaxing the surface, the GA will not act to modify the input values. This procedure holds the potential to resolve remaining questions around the dynamics and processes under relevant reaction conditions.

The wealth of new and detailed data now available from in particular high-pressure XPS on the temperature- and pressure-dependent coverages under reaction conditions of the highly relevant catalytic reactions above can now be expected to provide the impetus to further develop our understanding of the surface in heterogeneous catalysis. Here, capitalistic chemistry holds the key to the energetics.
